# Supplementary material for: Integrating whole genome and transcriptome sequencing to characterize the genetic architecture of isoform variation
Source: Nat Commun. 2025 Nov 22;16:10615. doi: 10.1038/s41467-025-64336-8 (PMC12660940; doi:10.1038/s41467-025-64336-8)
Supplement: Supplementary file 1 — Supplementary Information [file 41467_2025_64336_MOESM1_ESM.pdf]

Supplementary Information

Integrating Whole Genome and Transcriptome Sequencing to Characterize the Genetic Architecture of Isoform Variation and its Implications for Health and Disease

Liu, Joehanes, and Ma, et al.

SUPPLEMENTARY METHODS

The Jackson Heart Study (JHS)

We analyzed 1,012 participants from the Jackson Heart Study (JHS) who self-identified as African American (**Supplementary Table 1**).[22] Study protocols for participant examinations and genetic material collection were approved by the Institutional Review Board at the University of Mississippi Medical Center (UMMC). All participants provided written informed consent for genetic research, and all procedures were conducted in accordance with relevant ethical guidelines and regulations.

In JHS, peripheral blood mononuclear cells (PBMC) samples from 1,012 samples were used for RNA extraction at the University of Washington Northwest Genomics Center (NWGC), an NHLBI TOPMed program reference laboratory. Detailed information on library construction and processing was previously described.[19] Briefly, total RNA was scaled to 7.5ng/ul (total volume of 50ul) using the Perkin Elmer Janus II Workstation for library construction. The TruSeq Stranded mRNA Kit (Illumina, cat# RS-122-2103) was used for poly-A selection and cDNA synthesis. Successful constructed libraries were normalized and pooled prior to sequencing. RNA sequencing was performed at the NWGC, following a standard protocol.[25] The same analytical pipeline used in the Framingham Heart Study (FHS) was applied by the same analyst, as described in the main text. The JHS samples were normalized using the same approach applied to the FHS and WHI samples to ensure consistency.

The JHS conducted RNA-seq for 1,012 African-American participants who also had WGS.[30] We analyzed the JHS data using the isoform ratio method in 1,010 JHS participants after removing two participants with possible sample identification

problems. The methods for calculation of isoform ratio and association of genetic variants with isoform ratios were the same as those described in the main text. The replication rate was calculated as the ratio of the number of *cis*-sQTL-isoform pairs with p < 1e-4 and consistent effects (i.e., the same directionality for beta estimates) between the FHS discovery sample and JHS.

TOPMed RNA-seq pipeline harmonization summary

TOPMed used a consistent pipeline to harmonize RNA-seq data for TOPMed cohorts. Details can be found at [gtex-pipeline/TOPMed_RNAseq_pipeline.md at master · broadinstitute/gtex-pipeline · GitHub]

The STAR processing is detailed as below.

```
STAR --runMode alignReads \
    --runThreadN 8\
    --genomeDir ${star_index} \
    --twopassMode Basic \
    --outFilterMultimapNmax 20 \
    --alignSJoverhangMin 8 \
    --alignSJDBoverhangMin 1 \
    --outFilterMismatchNmax 999 \
    --outFilterMismatchNoverLmax 0.1 \
    --alignIntronMin 20 \
    --alignIntronMax 1000000 \
    --alignMatesGapMax 1000000 \
    --outFilterType BySJout \
    --outFilterScoreMinOverLread 0.33 \
    --outFilterMatchNminOverLread 0.33 \
    --limitSjdbInsertNsj 1200000 \
    --readFilesIn ${fastq1} ${fastq2} \
    --readFilesCommand zcat \
    --outFileNamePrefix ${sample_id} \
    --outSAMstrandField intronMotif \
    --outFilterIntronMotifs None \
    --alignSoftClipAtReferenceEnds Yes \
    --quantMode TranscriptomeSAM GeneCounts \
    --outSAMtype BAM Unsorted \
    --outSAMunmapped Within \
    --genomeLoad NoSharedMemory \
    --chimSegmentMin 15 \
    --chimJunctionOverhangMin 15 \
```

```
--chimOutType Junctions WithinBAM SoftClip \
--chimMainSegmentMultNmax 1 \
--outSAMattributes NH HI AS nM NM ch \
--outSAMattrRGline ID:rg1 SM:sm1
```

Prediction of protein structures by Google AlphaFold 3.0

We used Google AlphaFold 3.0 to predict protein structures[3,4] for the splicing variant rs12898397 [T>C] in *ULK3* [unc-51 like kinase 3]) as an example of how such variants might affect protein structure (**Supplementary Materials**). rs12898397 [T>C] is located in the COOH-terminal region of ULK3 (NM_001411082.1). To confirm the structure, we first aligned it with the reference 4WZX crystal structure from the Protein Data Bank[5] using the PyMOL Molecular Graphics System (Version 3.0.4, Schrödinger, LLC).[3] The 4WZX fragment contains 87-amino acids (aa): TSARDLLREMARDKPRLLAALEVASAAMAKEEAAGGEQDALDLYQHSLGELLLLLA AEPPGRRRELLHTEVQNLMARAEYLKEQvkM

For comprehensive evaluation, we predicted the structures of the three fragments of the COOH-terminal region of ULK3, in response to rs12898397 (T>C), using Google AlphaFold 3.0:

1) The fragment with the same aa sequence as in the 4WZX crystal structure
2) The fragment with 200-aa, with an 87-aa region upstream and 26aa downstream of the 4WZX sequence (the 4wzx sequence in uppercases):

ehmpsgeslgratalvvqavkkdqegdsaaalslyckaldffvpalhyevdaqrkeaikakvgqyvsraeelkaivs ssnqallrqgTSARDLLREMARDKPRLLAALEVASAAMAKEEAAGGEQDALDLYQHSLG ELLLLLAAEPPGRRRELLHTEVQNLMARAEYLKEQVKMresrweadtldkeglsesvrssctlq

3) The fragment with 173-aa, with 5-aa of NH2 terminal and 22-aa COOH terminal being removed from the 200aa-model due to low confidence of their structures

The overlay of these structures was visualized in PyMOL. The structural differences were evaluated using root-mean-square deviation (RMSD) values. An RMSD of 1.0 or lower Indicates nearly identical in conformations, while RMSD of 3.0 or higher suggests significant structural differences.[6]

SUPPLEMENTARY Data Description

Supplementary Data 1. Comparison of major characteristics between the 2015 paper and the present study
Supplementary Data 2. Characteristics of the Jackson Heart Study
Supplementary Data 3. Variants with MAF ≥0.01: significant sentinel cis-irQTL-isoform pairs (n=14,056, p<5e-8) in the discovery and replication samples.
Supplementary Data 4. Variants with MAF ≥0.01: significant sentinel trans-sQTL-isoform pairs (n=2,999, p<1.5e-13) in the discovery and replication samples
Supplementary Data 5. Rare variants with MAF < 0.01: significant sentinel cis-sQTL-isoform pairs (n=3,102, p<5e-8) in the discovery and replication samples
Supplementary Data 6. Replication rate of the 14,056 cis-sQTL-isoform pairs
Supplementary Data 7. Comparison of the source and processing of RNA samples between the FHS and JHS cohorts
Supplementary Data 8. Replication of 2015 paper most significant irQTL-gene pairs with cis-irQTL-gene pairs
Supplementary Data 9. Replication rate of 2,999 trans-irQTL-isoform pairs
Supplementary Data 10. Replication rate of 3,102 rare cis-irQTL-isoform pairs.
Supplementary Data 11. Comparison of eQTLs and irQTLs
Supplementary Data 12. Enrichment analysis of significant cis-irQTLs with GWAS SNPs
Supplementary Data 13. Enrichment analysis of top trans-irQTLs with GWAS SNPs
Supplementary Data 14. Detected transcripts for OAS1, ULK3, and CNN2 genes in the FHS
Supplementary Data 15. Mendelian randomization analysis of isoforms in three genes to cardiovascular disease traits
Supplementary Data 16. Relevant eQTLs and irQTLs in ULK3 gene
Supplementary Data 17. Compare methods: isoform ratio, exon-level QTL, and LeafCutter QTL
Supplementary Data 18. GWAS datasets used for MR analysis

SUPPLEMENTARY FIGURES

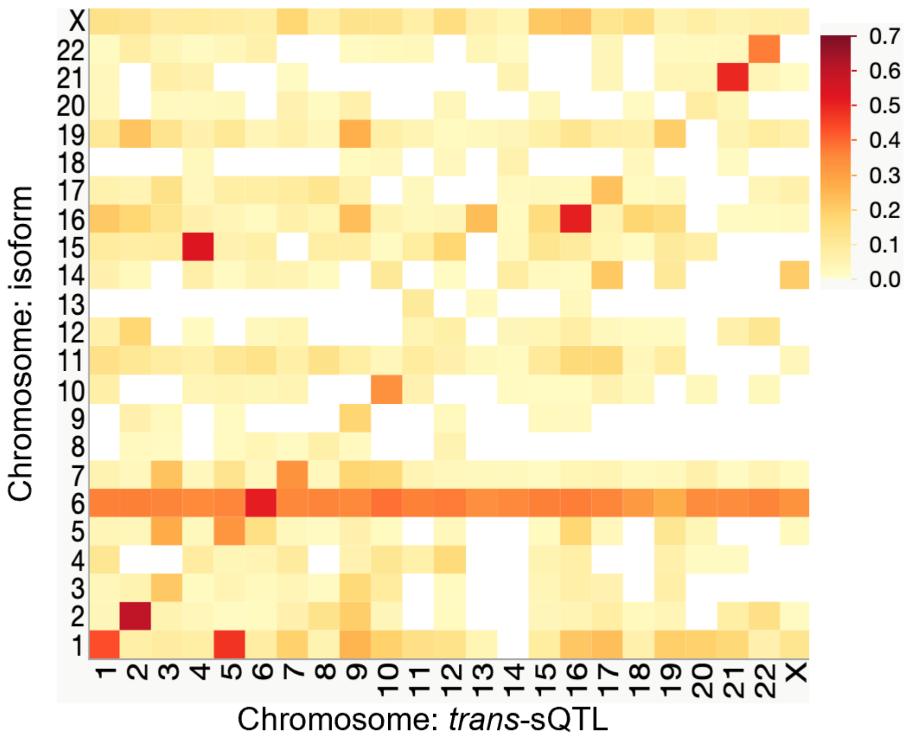

Supplementary Figure 1. Heatmap of maximum R^2 values for *trans*-sQTL-isoform associations by chromosome locations. Maximum R^2 values were calculated across all *trans*-sQTL–isoform pairs located on the same chromosome combination.

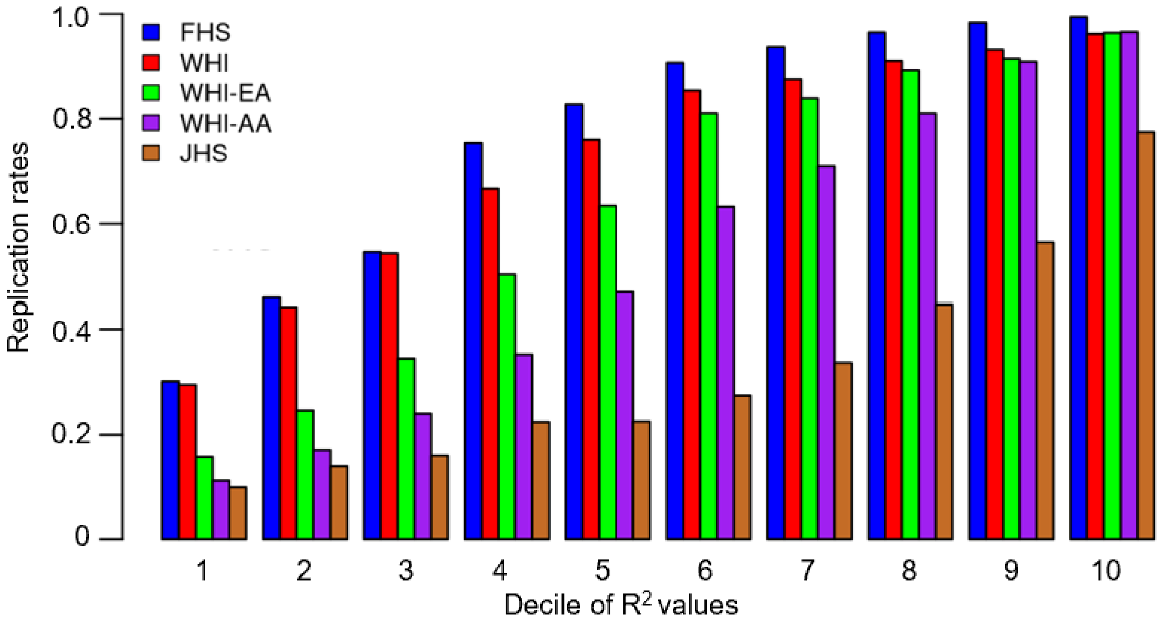

Supplementary Figure 2. Replication of sentinel *cis*-irQTLs in relation to their associated isoforms. We identified 11,425 common variants (MAF ≥ 0.01) as sentinel *cis*-irQTLs for 10,883 isoforms, comprising 4,971 sGenes in the discovery FHS sample (n=2,622). Internal replication was performed in an additional Framingham Heart Study (FHS) sample (n=1,094), and external replication was conducted in Women's Health Initiative (WHI) (n=2,005) and Jackson Heart Study (JHS) (n=1,010) samples. Separate analyses were also carried out in White American (WHI-EA) and African American (WHI-AA) participants in WHI. An association of a sentinel *cis*-sQTL with its isoform ratio was considered replicated if its $P < 1e-4$ in the replication sample and showed the same direction of effect as in the discovery sample. Replication rate was defined as the proportion of discovered pairs that met these criteria in the replication sample. Replication rates (y-axis) of the sentinel *cis*-sQTL increases with R^2 (x-axis), the proportion of variance of the corresponding isoform ratio explained by the irQTLs. The average replication rate was 76.7% in FHS, 72.4% in WHI, and 34.2% in JHS. The average replication rate was 63.1% in WHI-EA and 53.7% in WHI-AA.

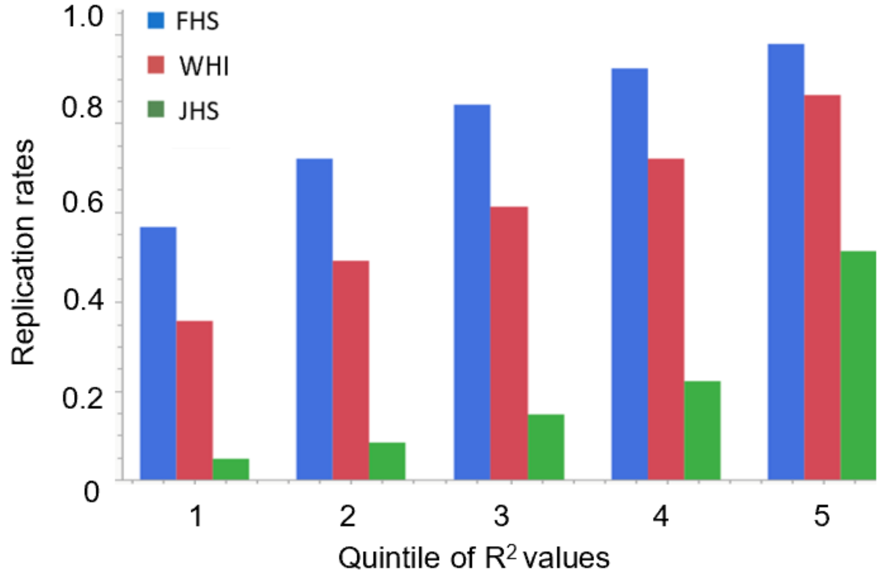

Supplementary Figure 3. Replication of common sentinel *trans*-irQTLs in relation to their associated isoforms. We identified 2,999 sentinel *trans*-irQTLs-isoform pairs (p < 1.5e-13), involving 1,870 *trans*-irQTLs and 1,084 isoforms across 590 sGenes in the discover sample (n=2,622). Internal replication was performed in an additional FHS sample (n=1,094), and replication in WHI (n=2,005) and JHS (n=1,010) samples. An association of a sentinel *trans*-sQTL with its isoform ratio was considered replicated if its P < 1e-4 in the replication sample and showed the same direction of effect as in the discovery sample. Replication rate was defined as the proportion of discovered pairs that met these criteria in the replication sample. Replication rates (y-axis) of the sentinel *cis*-sQTL increases with R^2 (x-axis) the proportion of variance of the corresponding isoform ratio explained by the irQTLs. The average replication rates were 80.5% in FHS, 60.8% in WHI, and 20.2% in JHS.

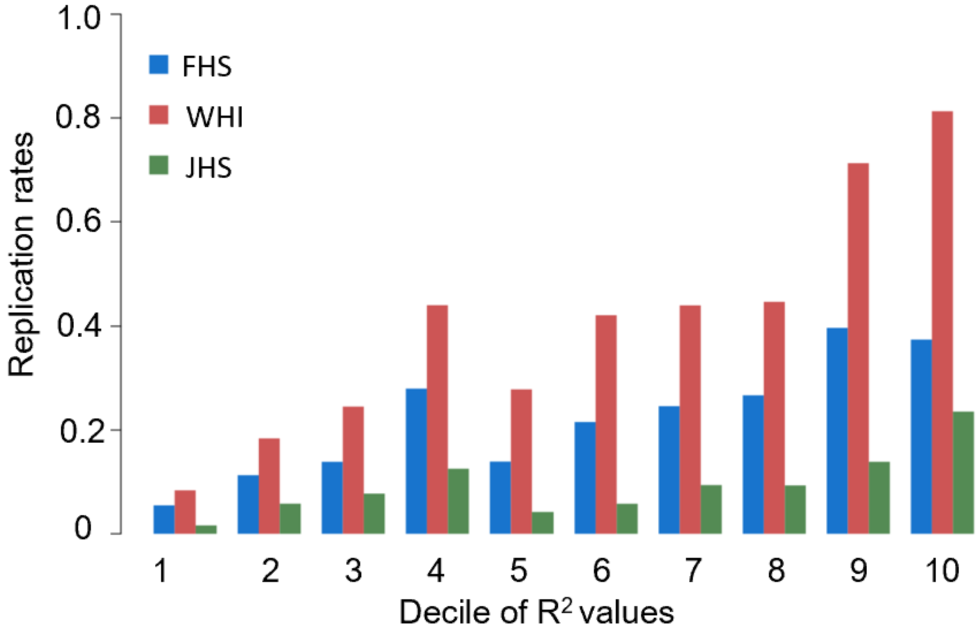

Supplementary Figure 4. **Replication of rare sentinel *cis*-irQTLs in relation to their associated isoforms.** We identified 2,327 sentinel rare variants as *cis*-irQTLs associated with 2,467 isoforms in the discovery FHS sample (n=2,622). Internal replication was performed in an independent FHS sample (n=1,094), and external replication was conducted in WHI (n=2,005) and JHS (n=1,010) samples. A sentinel irQTL-isoform ratio association was considered replicated if its $P < 1\times10^{-4}$ in the replication sample and showed the same direction of effect as in the discovery sample. Replication rate was defined as the proportion of discovered pairs that met these criteria in the replication sample. Replication rates (y-axis) of the sentinel *cis*-sQTL increases with R^2 (x-axis), the proportion of variance of the corresponding isoform ratio explained by the irQTLs. The average replication rates were 22.2% in FHS, 40.7% in WHI, and 9.4% in JHS.

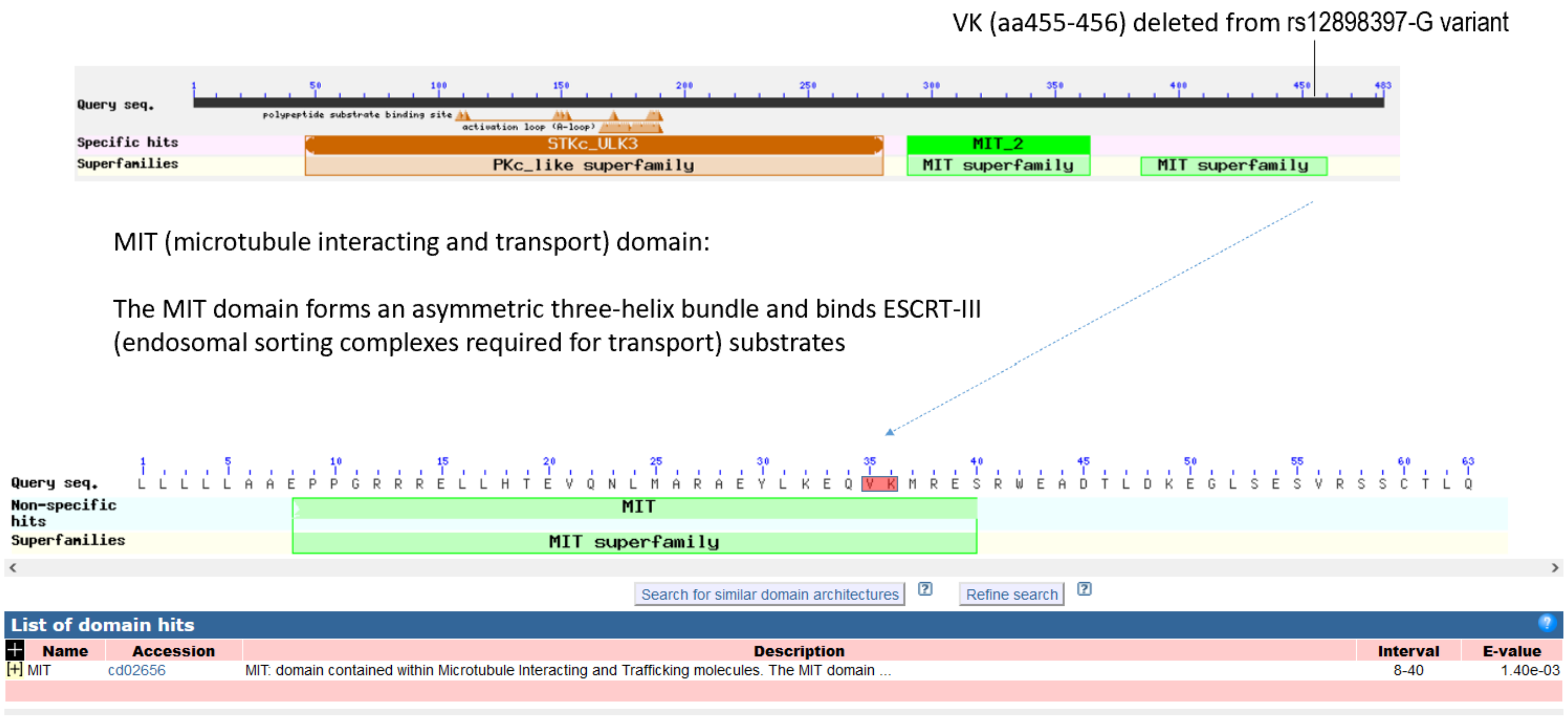

Supplementary Figure 5. rs12898397-G>A region. MIT, microtubule interacting and transport domain. The rs12898397-G is the sentinel *cis*-sQTL for transcript ENST00000440863.7 (*ULK3*-201) which produces a full-length protein with 472 amino acids, whereas isoform ENST00000569437.5 (*ULK3*-220) produces a truncated protein with two amino acids ("VK") missing at the 5' of the 14th exon (**Figure 5A**).

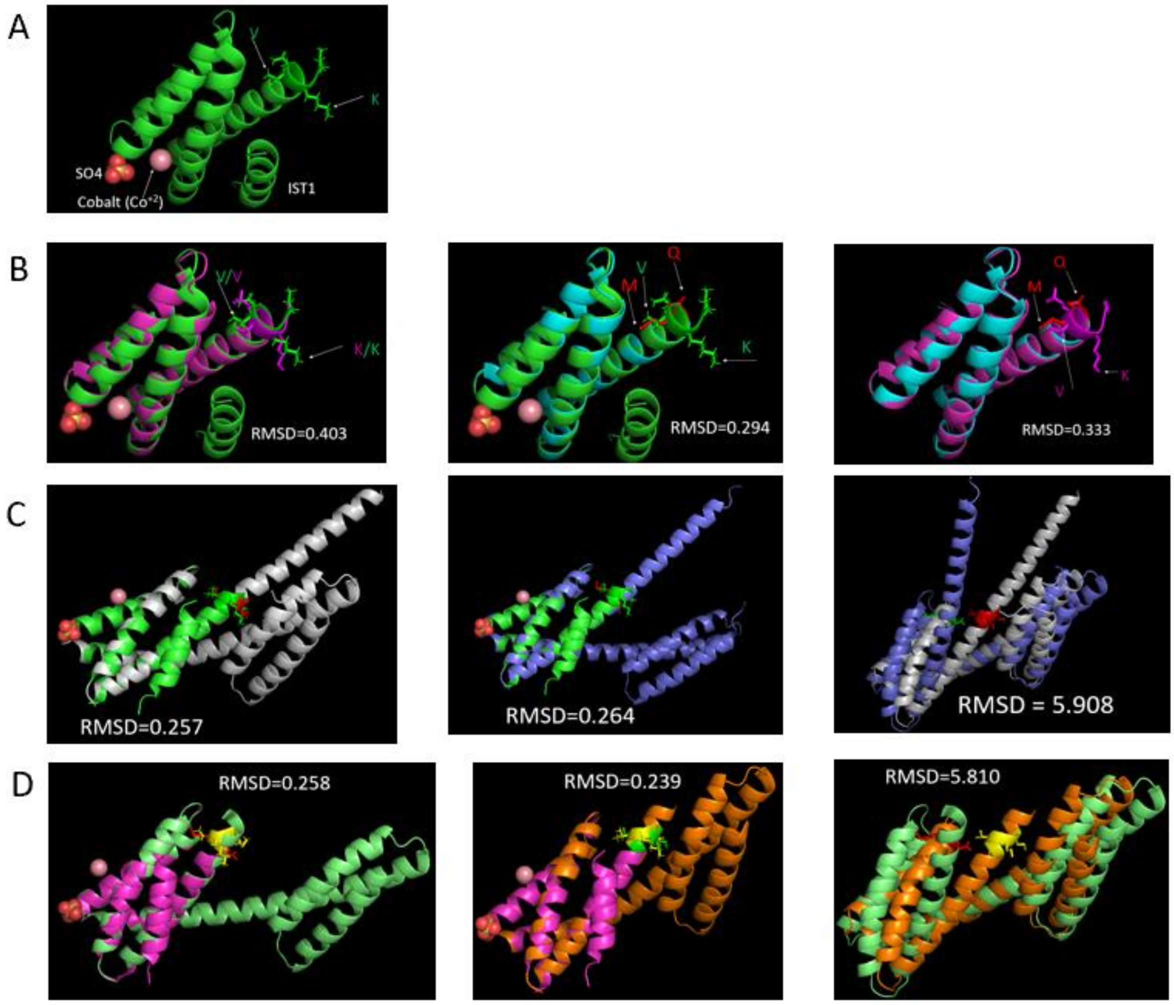

Supplementary Figure 6. Pymol visualization of AlphaFold 3.0-predicted COOH-terminal domains of ULK3 protein variants with and without "VK" amino acids (aa). The structural differences were evaluated using root-mean-square deviation (RMSD) values. An RMSD of 1.0 or lower Indicates nearly identical in conformations, while RMSD of 3.0 or higher suggests significant structural differences

A. The crystal structure of the ULK3 COOH-terminal region, 4WZX (87-aa), complexed with IST1, sulfate, and cobalt ions, is shown. The 87-aa sequence is: TSARDLLREMARDKPRLLAALEVASAAMAKEEAAGGEQDALDLYQHSLGELLLLLA AEPPGRRRELLHTEVQNLMARAEYLKEQvkM

B. Alignment of 4WZX with the ULK3 region containing the same aa sequence shows consistent configurations, with RMSD = 0.403 (with “VK”) on the left, and RMSD = 0.294 (without “VK”) in the middle. The sequences of the ULK3 region with and without “VK” show similar conformations (RMSD = 0.333).

C. The 87-aa region upstream and 26aa downstream of the 4WZX sequence are added (the 4wzx sequence in capital case):

ehmpsgeslgratalvvqavkkdqegdsaaalslyckaldffvpalhyevdaqrkeaikakvgqyvsraeelkaivs ssnqallrqgTSARDLLREMARDKPRLLAALEVASAAMAKEEAAGGEQDALDLYQHSLG ELLLLLAAEPPGRRRELLHTEVQNLMARAEYLKEQVKMresrweadtldkeglsesvrssctlq

Alignment of 4WZX with the 200-aa AlphaFold COOH-terminal model, with and without "VK," shows RMSD values of 0.257 (with "VK") and 0.264 (without "VK"). The 200-aa with "VK" and 198-aa without "VK" show different conformations (RMSD = 5.908).

D. The 173-aa model removes 5-aa of the NH2-terminal and 22-aa of the COOH-terminal low-confidence segments (Supplementary Figure 7). Alignment of 4WZX with the 173-aa AlphaFold model shows RMSD values of 0.258 (with "VK") and 0.239 (without "VK") to confirm confirming the structural similarity between modeled sequences and to the known crystal structure 4WZX. The 173-aa model with "VK" and 171-aa without "VK" shows significant conformational differences (RMSD = 5.810), indicating substantial structural differences resulting from the two-codon skipping event.

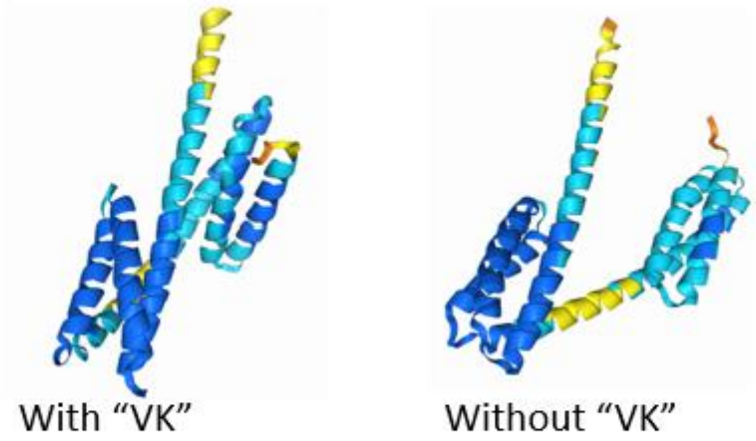

Supplementary Figure 7. AF3 prediction of ULK3 200-aa COOH-terminal structure of ULK3 protein with and without “VK”. The 22-aa in COOH-terminal terminal and 5-aa of the NH2-terminal show low confidence in yellow or orange colors. There is also a fragment of low confidence region in the middle of this fragment.

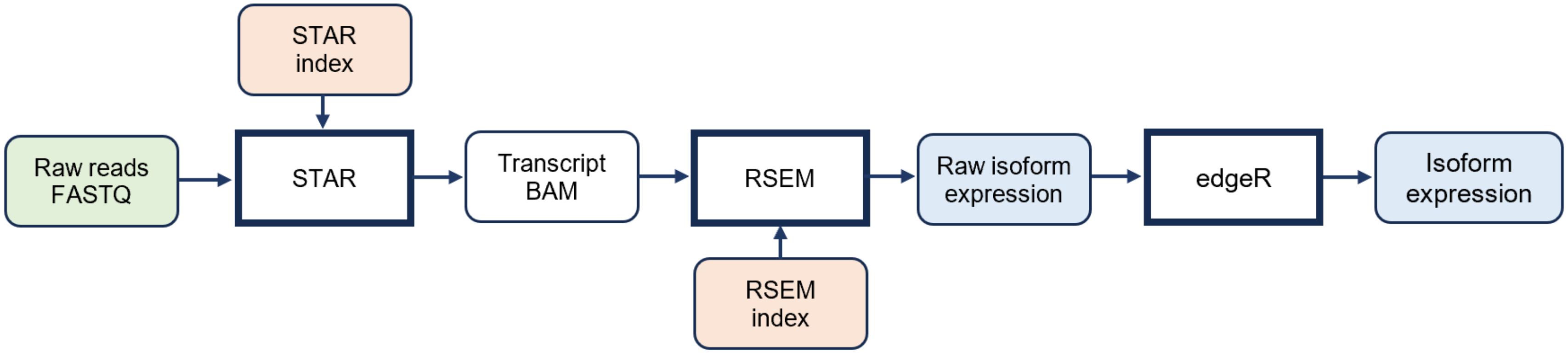

References

Reference genome	GRCh38
Transcript annotation	GENCODE 30

Pipeline components

Alignment	STAR v2.6.1d
Transcript expression	RSEM v1.3.1
Normalization	edgeR

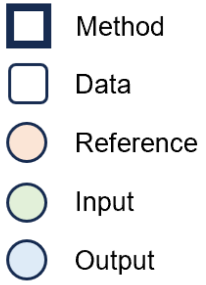

Supplementary Figure 8. Flowchart of processing and quality control of RNA-seq data in TOPMed. (https://topmed.nhlbi.nih.gov/sites/default/files/TOPMed_RNAseq_pipeline_flowchart_COREyr3.pdf).

Reference

1. Consortium, G.T. The GTEx Consortium atlas of genetic regulatory effects across human tissues. *Science* **369**, 1318-1330 (2020).
2. Garrido-Martin, D., Borsari, B., Calvo, M., Reverter, F. & Guigo, R. Identification and analysis of splicing quantitative trait loci across multiple tissues in the human genome. *Nat Commun* **12**, 727 (2021).
3. Abramson, J. *et al.* Accurate structure prediction of biomolecular interactions with AlphaFold 3. *Nature* **630**, 493-500 (2024).
4. Jumper, J. *et al.* Highly accurate protein structure prediction with AlphaFold. *Nature* **596**, 583-589 (2021).
5. Berman, H.M. *et al.* The Protein Data Bank. *Nucleic Acids Res* **28**, 235-42 (2000).
6. Magala, P., Klevit, R.E., Thomas, W.E., Sokurenko, E.V. & Stenkamp, R.E. RMSD analysis of structures of the bacterial protein FimH identifies five conformations of its lectin domain. *Proteins* **88**, 593-603 (2020).
