# Supplementary material for: Integrating whole genome and transcriptome sequencing to characterize the genetic architecture of isoform variation
Source: Nat Commun. 2025 Nov 22;16:10615. doi: 10.1038/s41467-025-64336-8 (PMC12660940; doi:10.1038/s41467-025-64336-8)
Supplement: Supplementary file 2 — Description of Additional Supplementary Files [file 41467_2025_64336_MOESM2_ESM.pdf]

Description of Additional Supplementary Files

Supplementary Data 1: Comparison of major characteristics between the 2015 paper and the present study

Supplementary Data 2: Characteristics of the Jackson Heart Study

Supplementary Data 3: Variants with MAF ≥0:01: significant sentinel cis-irQTL-isoform pairs (n=14,056, p<5e-8) in the discovery and replication samples

Supplementary Data 4: Variants with MAF ≥0:01: significant sentinel trans-sQTL-isoform pairs (n=2,999, p<1:5e-13) in the discovery and replication samples

Supplementary Data 5: Rare variants with MAF < 0:01: significant sentinel cis-sQTL-isoform pairs (n=3,102, p<5e-8) in the discovery and replication samples

Supplementary Data 6: Replication rate of the 14,056 cis-sQTL-isoform pairs

Supplementary Data 7: Comparison of the source and processing of RNA samples between the FHS and JHS cohorts

Supplementary Data 8: Replication of 2015 paper most significant irQTL-gene pairs with cis-irQTL-gene pairs

Supplementary Data 9: Replication rate of 2,999 trans-irQTL-isoform pairs

Supplementary Data 10: Replication rate of 3,102 rare cis-irQTL-isoform pairs

Supplementary Data 11: Comparison of eQTLs and irQTLs

Supplementary Data 12: Enrichment analysis of significant cis-irQTLs with GWAS SNPs

Supplementary Data 13: Enrichment analysis of top trans-irQTLs with GWAS SNPs

Supplementary Data 14: Detected transcripts for OAS1, ULK3, and CNN2 genes in the FHS

Supplementary Data 15: Mendelian randomization analysis of isoforms in three genes to cardiovascular disease traits

Supplementary Data 16: Relevant eQTLs and irQTLs in ULK3 gene

Supplementary Data 17: Compare methods: isoform ratio, exon-level QTL, and LeafCutter QTL

Supplementary Data 18: GWAS datasets used for MR analysis
