# Supplementary material for: Integrating whole genome and transcriptome sequencing to characterize the genetic architecture of isoform variation
Source: Nat Commun. 2025 Nov 22;16:10615. doi: 10.1038/s41467-025-64336-8 (PMC12660940; doi:10.1038/s41467-025-64336-8)
Supplement: Supplementary file 4 — Reporting Summary [file 41467_2025_64336_MOESM4_ESM.pdf]

nature portfolio

Corresponding author(s): Chunyu Liu
Roby Joehanes
Daniel Levy

Last updated by author(s): Jul 30, 2025

Reporting Summary

Nature Portfolio wishes to improve the reproducibility of the work that we publish. This form provides structure for consistency and transparency in reporting. For further information on Nature Portfolio policies, see our Editorial Policies and the Editorial Policy Checklist.

Statistics

For all statistical analyses, confirm that the following items are present in the figure legend, table legend, main text, or Methods section.

n/a	Confirmed	
☐	☒	The exact sample size (n) for each experimental group/condition, given as a discrete number and unit of measurement
☒	☐	A statement on whether measurements were taken from distinct samples or whether the same sample was measured repeatedly
☐	☒	The statistical test(s) used AND whether they are one- or two-sided<br>*Only common tests should be described solely by name; describe more complex techniques in the Methods section.*
☐	☒	A description of all covariates tested
☐	☒	A description of any assumptions or corrections, such as tests of normality and adjustment for multiple comparisons
☐	☒	A full description of the statistical parameters including central tendency (e.g. means) or other basic estimates (e.g. regression coefficient) AND variation (e.g. standard deviation) or associated estimates of uncertainty (e.g. confidence intervals)
☐	☒	For null hypothesis testing, the test statistic (e.g. F, t, r) with confidence intervals, effect sizes, degrees of freedom and P value noted<br>*Give P values as exact values whenever suitable.*
☒	☐	For Bayesian analysis, information on the choice of priors and Markov chain Monte Carlo settings
☒	☐	For hierarchical and complex designs, identification of the appropriate level for tests and full reporting of outcomes
☐	☒	Estimates of effect sizes (e.g. Cohen's d, Pearson's r), indicating how they were calculated

Our web collection on statistics for biologists contains articles on many of the points above.

Software and code

Policy information about availability of computer code

Data collection	Summary data for several figures (Figs 2A, 2B, 3A, 3B, 3C, 4D, 5B, 5G, 6B) are available in https://figshare.com/account/home#/projects/256376. Source data are also provided in the Supplementary Data accompanying this paper. All summary statistics for irQTLs with P < 1e-4 are available at https://biodatacatalyst.nhlbi.nih.gov/ with the accession number phs001974.v8.p1.
Data analysis	All statistical analyses were conducted using R (version 4.4.0) or JMPTM (version 18.1.1). Codes to generate the figures (Figs 2A, 2B, 3A, 3B, 3C, 4D, 5B, 5G, 6B) are available in https://figshare.com/account/home#/projects/256376.

For manuscripts utilizing custom algorithms or software that are central to the research but not yet described in published literature, software must be made available to editors and reviewers. We strongly encourage code deposition in a community repository (e.g. GitHub). See the Nature Portfolio guidelines for submitting code & software for further information.

Data

Policy information about availability of data

All manuscripts must include a data availability statement. This statement should provide the following information, where applicable:

- Accession codes, unique identifiers, or web links for publicly available datasets
- A description of any restrictions on data availability
- For clinical datasets or third party data, please ensure that the statement adheres to our policy

We used previously collected data from three cohorts, including the Framingham Heart Study, Jackson Heart Study, ans Women's Health Initiative. The whole genome sequencing (WGS), RNA sequencing (RNA-seq), and phenotypic data from the Framingham Heart Study (FHS), the Jackson Heart Study (JHS), and the Women's Health Study (WHS) have been deposited in the dbGaP database under accession codes, phs000007.v32.p13 (FHS) [https://www.ncbi.nlm.nih.gov/projects/gap/cgi-bin/study.cgi?study_id=phs000007.v34.p15], phs000964 (JHS) [https://www.ncbi.nlm.nih.gov/projects/gap/cgi-bin/study.cgi?study_id=phs000964.v5.p1], phs001237 (WHI) [https://www.ncbi.nlm.nih.gov/projects/gap/cgi-bin/study.cgi?study_id=phs001237.v3.p1]. The WGS, RNA-seq, and phenotypic data from all cohorts used in this study are available under restricted access to ensure confidentiality and protect participant privacy. Access can be obtained by an approved IRB and ancillary study proposal. The approval process range from 3–7 weeks across the FHS, JHS, and WHI.

Research involving human participants, their data, or biological material

Policy information about studies with human participants or human data. See also policy information about sex, gender (identity/presentation), and sexual orientation and race, ethnicity and racism.

Reporting on sex and gender	This study included 54.5% women in FHS, 100% in WHI, and 64.4% in JHS, based on self-reported sex (Table and Supplementary Data 2). Sex distribution was not part of the study design, as the cohorts were previously established as population-based studies. No sex-specific analyses were performed.
Reporting on race, ethnicity, or other socially relevant groupings	Self-identified race was included.
Population characteristics	We described age, self-reported sex in Table 1. We included 3,716 participants (n=2,622 for initial discovery and n=1.094 for internal replication), mostly self-identified American Whites primarily from the FHS Offspring and Third Generation cohorts (Table 1).59,60 Blood samples for RNA-seq were collected at the ninth examination cycle (2011–2014) of the Offspring cohort and the second examination cycle (2008–2011) of the Third Generation cohort (Table 1). In WHI, blood samples for RNA-seq were collected during a follow-up examination (2012–2013) conducted as part of the Long Life Study (LLS), a sub-study within the larger WHI cohort.23 The LLS repeated selected components of the original WHI physical examination and collected blood samples from approximately 8,000 participants across the United States.61 A total of 2,005 WHI participants were included in the present analysis, comprising 1,000 Whites, 918 African Americans, and 87 participants self-identified as other race/ethnicity groups. We did not exclude any participants based on genetic similarity to reference panels such as 1000G. We also analyze 1,010 self-identified African American participants from JHS (Supplemental Materials). Protocols for participant examinations and genetic material collection were approved by the Institutional Review Boards at the respective research sites. All participants provided written, informed consent for genetic studies. All research was performed in accordance with relevant guidelines and regulations.
Recruitment	These are previously collected data from population cohorts.
Ethics oversight	All research was conducted in accordance with relevant ethical guidelines and regulations. Study protocols were approved by the Institutional Review Boards (IRBs) at participating sites: the Boston Medical Center and Boston University Medical Campus IRB (FHS); Jackson State University, Tougaloo College, and the University of Mississippi Medical Center IRBs (JHS); and the IRBs of all participating institutions, with oversight from the Fred Hutchinson Cancer Research Center IRB (WHI). All participants provided written informed consent for genetic studies.

Note that full information on the approval of the study protocol must also be provided in the manuscript.

Field-specific reporting

Please select the one below that is the best fit for your research. If you are not sure, read the appropriate sections before making your selection.

☒ Life sciences ☐ Behavioural & social sciences ☐ Ecological, evolutionary & environmental sciences

For a reference copy of the document with all sections, see nature.com/documents/nr-reporting-summary-flat.pdf

Life sciences study design

All studies must disclose on these points even when the disclosure is negative.

Sample size	We used existing data that are available to us. We used 2622 Framingham Heart Study participants as discovery sample, followed by replication in an additional FHS sample (n=1094) and Women's health Initiative (n=2005).

Data exclusions	We excluded samples if they were identified as data swap. No other exclusions were applied
Replication	The discovery analysis was conducted in the Framingham Heart Study discovery sample (n=2622), followed by replication in an additional FHS sample (n=1094) and Women's health Initiative (n=2005). No repeated measures were generated.
Randomization	Not applicable
Blinding	Not applicable

Reporting for specific materials, systems and methods

We require information from authors about some types of materials, experimental systems and methods used in many studies. Here, indicate whether each material, system or method listed is relevant to your study. If you are not sure if a list item applies to your research, read the appropriate section before selecting a response.

Materials & experimental systems

n/a	Involved in the study
☒	☐ Antibodies
☒	☐ Eukaryotic cell lines
☒	☐ Palaeontology and archaeology
☒	☐ Animals and other organisms
☒	☐ Clinical data
☒	☐ Dual use research of concern
☒	☐ Plants

Methods

n/a	Involved in the study
☒	☐ ChIP-seq
☒	☐ Flow cytometry
☒	☐ MRI-based neuroimaging

Plants

Seed stocks	Not applicable
Novel plant genotypes	Not applicable
Authentication	Not applicable
